# Should we leave paediatric emergency triage to artificial intelligence? A comparison of ChatGPT 4o and Grok 3

**DOI:** 10.3389/fped.2026.1739217

**Published:** 2026-02-17

**Authors:** Emre Aygun, Aysenur Imdat, Nazan Dalgic

**Affiliations:** 1 Department of Pediatrics, Şişli Hamidiye Etfal Training and Research Hospital, University of Health Sciences, Istanbul, Türkiye; 2 Department of Pediatric Infectious Diseases, Şişli Hamidiye Etfal Training and Research Hospital, University of Health Sciences, Istanbul, Türkiye

**Keywords:** artificial intelligence, emergency service, hospital, pediatrics, triage

## Abstract

**Background:**

The growing number of patients in paediatric emergency departments requires fast and precise triage assessments. The implementation of large language models faces obstacles due to their limited interpretability. We aimed to compare the performance of ChatGPT 4o and Grok 3 with that of nurses and physicians in paediatric emergency triage.

**Methods:**

This prospective observational study evaluated paediatric emergency patients presenting to our paediatric emergency department between March and April 2025. Demographic data, chronic disease status, presenting complaints, and vital signs were documented. Patients were triaged according to ESI criteria by nurses, paediatric specialists (gold standard), ChatGPT 4o, and Grok 3. Inter-rater agreement was analysed using Cohen's kappa**. Cochran's Q and McNemar's tests were used for paired comparisons.**

**Results:**

A total of 1,505 paediatric emergency patients were included in the analysis. No ESI-1 cases were observed; therefore, critical patients were defined as ESI-2. Nurses achieved 53.1% (95% CI: 50.6–55.6) accuracy in triage assessments, while ChatGPT 4o achieved 76.1% (95% CI: 73.9–78.2) and Grok 3 achieved 47.0% (95% CI: 44.5–49.6) accuracy (Cochran's Q = 275.68, *p* < 0.001). ChatGPT 4o showed good agreement with physicians (*κ* = 0.69). For critical patient identification, sensitivity was 37.2% for nurses, 82.9% for ChatGPT 4o, and 97.7% for Grok 3; however, Grok 3 demonstrated substantial over-triage (36.3%) and low positive predictive value (37.2%). ChatGPT 4o achieved the lowest mean absolute ESI error (0.25 ± 0.45). Nurses' critical patient recognition improved from 28.3% to 59.5% (*p* < 0.01) for children with chronic illnesses.

**Conclusion:**

ChatGPT 4o achieved the most favourable balance of sensitivity and specificity. The superior performance of nurses in recognising critically ill patients with chronic diseases suggests that AI systems should augment nursing expertise rather than replace it.

## Introduction

The world's healthcare systems now depend heavily on emergency departments because they handle rising patient numbers while facing limited resources (1). The fast and precise identification of children in pediatric emergency departments requires special attention because their body systems operate differently from adults (2). The five-level Emergency Severity Index (ESI) stands as the standard triage system which medical professionals accept because it demonstrates both reliability and validity (3). The assessment methods used in traditional triage systems depend on nurse expertise yet produce variable results especially when hospitals experience high patient volumes (4). Healthcare decision support through artificial intelligence and machine learning technologies has shown promising capabilities for emergency department triage since their rapid development in recent years (5). Large language models deployed in pediatric emergency departments deliver improved diagnostic accuracy and predictive performance to the system. AI systems help healthcare professionals make rapid decisions through their ability to analyze multiple clinical parameters at high speed. The implementation process encounters specific challenges because of limited training data for children and the requirement to accommodate distinct physiological patterns that exist across different age groups (6, 7).

The current literature evaluates AI-based triage system technical capabilities but lacks sufficient evidence about their effectiveness and reliability when used in actual clinical settings (1). Research findings about AI model performance compared to nurse and physician assessments in pediatric patients remain scarce because of insufficient prospective studies (2). The research lacks sufficient data about how artificial intelligence systems perform triage tasks for patients with chronic illnesses within specific age groups (7). The literature contains mostly retrospective single-center studies about artificial intelligence-based triage systems but their ability to work across different patient groups and clinical environments remains unclear (4). The literature lacks information about how nurses experience ESI application variations and how AI systems handle these differences (3). The performance evaluation of new-generation language models including ChatGPT and Grok for pediatric emergency triage remains limited in the research community (6).

Healthcare organizations face particular obstacles when they use large language models (LLMs) because these systems operate through different mechanisms from conventional machine learning systems. The black box nature of LLMs prevents healthcare providers and patients from understanding their clinical reasoning because these models lack the interpretability which conventional predictive models provide through their structured datasets with established input-output connections (1, 4). The implementation of LLMs creates two essential security threats which endanger patient safety because these systems generate authentic yet medically inaccurate and fabricated information during urgent triage operations (6). The process of clinical information transfer to commercial AI platforms requires organizations to address data privacy and governance issues because it creates challenges regarding data storage duration and healthcare rule compliance and information reuse (2). The implementation of LLMs in clinical practice faces multiple operational difficulties because of system response times and unexpected AI outputs and healthcare staff must review all AI suggestions (5). The evaluation of LLMs for pediatric emergency triage needs solutions which solve these problems because children require special medical care due to their physiological vulnerability.

The main objective of this research investigates how ChatGPT 4o and Grok 3 artificial intelligence systems perform in ESI-based triage assessments against human nurses and physicians in pediatric emergency departments. Research findings show that ChatGPT-4 and other large language models demonstrate emergency triage capabilities through 76.6% accuracy performance better than conventional triage systems (8) and they show effective agreement with medical professionals in clinical environments (9). Research studies through meta-analysis show AI systems maintain reliable performance in triage decision-making because they achieve accuracy levels between 63% and 76% across various implementations (10). The research evidence supports our prediction that AI systems will achieve equal or superior results to those of nursing staff in total triage accuracy and will excel at detecting patients with severe conditions (ESI-2, as ESI-1 cases are rare in outpatient pediatric emergency settings) beyond nursing capabilities. We further hypothesize that nurses will achieve better triage outcomes for children with chronic diseases through their clinical experience while AI systems will maintain consistent performance regardless of chronic conditions. The research aims to demonstrate AI triage system advantages and disadvantages for pediatric emergency departments while developing evidence-based recommendations for clinical practice.

## Methods

### Study population and sample

This study was designed as a single-centre, prospective, observational study and was conducted at the Paediatric Emergency Department of Health Sciences University Sarıyer Hamidiye Etfal Training and Research Hospital between 26 March 2025 and 26 April 2025. In calculating the sample size, it was estimated that approximately 1,500 patients would be reached in one month, considering an average of 250–300 patient visits per day and after applying exclusion criteria. The initial evaluation used an assumed accuracy of 50% for the worst-performing assessor to estimate the required sample size, allowing calculation of 95% confidence intervals with a ± 2.5 percentage point margin of error. The inclusion criteria for the study were defined as being under 18 years of age and presenting to the emergency department as an outpatient.

**Figure 1:**
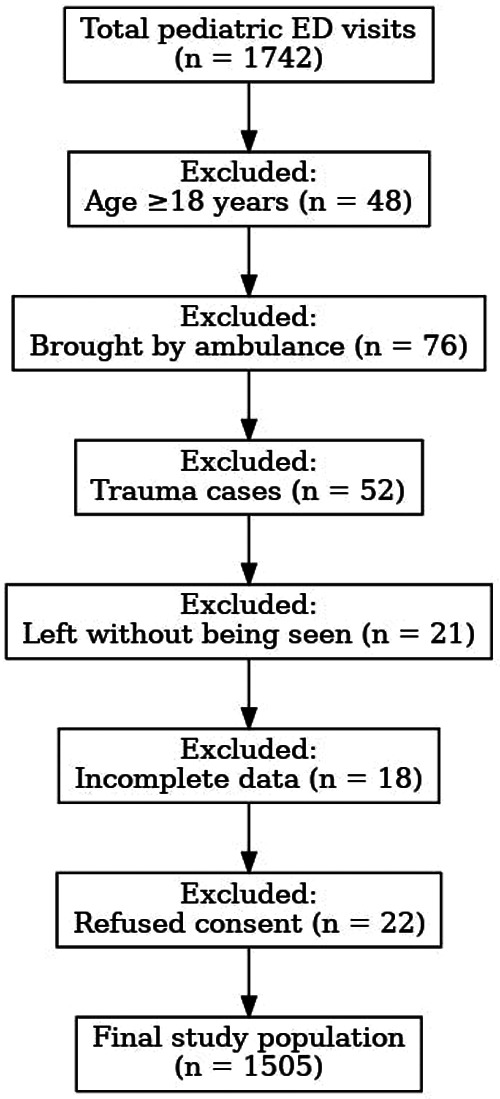
Patient selection flow chart: total pediatric emergency department (ED) visits, exclusion criteria, and final study population (*n* = 1,505).

The study excluded patients who arrived by ambulance because their assessment process deviated from typical triage procedures. The study excluded trauma patients because they need special triage methods and patients who left before being evaluated because their medical information was not complete. Exclusion criteria were defined as patients aged 18 years and older, those brought by ambulance, those who left without examination or without following medical advice, those presenting with trauma, those with incomplete information or data, and those who refused to participate in the study (Figure 1).

In the operational definitions of the variables, the presence of chronic disease was defined as the patient having any previously diagnosed and monitored systemic disease, critical patients were defined as those assessed as ESI-1 or ESI-2, over-triage was defined as an ESI score lower than that given by the physician assessment, and under-triage was defined as an ESI score higher than that given by the physician assessment. The primary outcome measure included patient disposition which resulted in either discharge or admission to a ward or intensive care unit.

The researchers selected ESI because it represents the most validated five-level triage system for emergency departments which shows reliable results when used with pediatric patients (3, 11). The START/JumpSTART system functions best for mass casualty situations but it lacks effectiveness in standard emergency department triage operations.

### Study procedures

During the data collection process, patients' demographic characteristics (age, gender), chronic disease status and type, time of presentation (weekday/weekend), presenting complaints and duration, vital signs (temperature, respiratory rate, oxygen saturation, heart rate, blood pressure), and disposition information (discharge, ward admission, intensive care admission) were recorded using a standard form.

For the triage assessment of patients, scoring was first performed by a team of paediatric emergency nurses with a minimum of 2 years of clinical experience according to the ESI 5th edition guidelines. Most nurses had finished their advanced triage education courses which went past their basic ESI training. Reassessment was performed by experienced paediatric specialists independently of the nurses' assessments using a blinded method. The authors were not included in the physician assessment group to avoid potential bias.

The physician ESI assessments took place right after nurse triage was initiated, within 15–30 min of when patients entered the facility. The assessment used all available information from patient arrival until triage completion which included their symptoms and vital signs and chronic health conditions and limited physical assessment results. Physicians did not have access to any laboratory results, imaging studies, or treatment responses at the time of ESI assignment. The method used reference standards which contained the same clinical data which nurses and AI systems could access to prevent verification bias from occurring. Physicians gained an advantage through their ability to observe patients directly while using their clinical expertise to make decisions which aligns with actual triage procedures in practice.

The physician assessments served as the gold standard because they combined ESI criteria with complete clinical evaluation which previous triage accuracy studies had validated (12). The routine nature of nurse triage in clinical settings does not match the comprehensive clinical understanding of physicians who work under less time constraints thus making physician assessments suitable for research reference standards. Although multiple physicians were involved in the assessments, formal inter-rater reliability assessment was not performed due to the independent and sequential nature of patient evaluations; this is acknowledged as a limitation of the study.

Physicians did not see the ESI scores given by nurses during the gold standard assessment. All nurses and physicians underwent standard training according to the ESI 5th edition guidelines prior to the study. For artificial intelligence assessments, ChatGPT 4o (OpenAI, model version gpt-4o-2024-05-13) and Grok 3 (xAI, version 3.0) were accessed via their official web interfaces during the period of 26 March to 26 April 2025. These systems were provided with standardised training specific to ESI criteria; the hospital's working conditions and physical characteristics were introduced in advance. Default inference parameters were used for both systems (temperature settings were not manually adjusted, as web interface access does not allow parameter modification). The AI systems evaluated each patient case only once to mimic actual clinical practice but they did not perform additional evaluations for stochastic variation analysis because of this acknowledged limitation. The AI system generated a single ESI level between 1 and 5 for all outputs which eliminated the need for human interpretation or data transformation. The research team excluded all cases where AI generated unclear or multiple answers because these responses did not fit the predefined analysis criteria. Triage decisions were based solely on patients' clinical characteristics; external factors such as emergency department workload or resource constraints were not included in the assessment; the same applied to the artificial intelligence systems.

### Intervention protocol

Four different evaluator groups were formed in the study: nurses, physicians (gold standard), ChatGPT 4o, and Grok 3. Clinical information for each patient was prepared in a standardised format in English. Patient data was fed to the AI systems in a single submission for each case to simulate real-time triage decision-making. The artificial intelligence systems were asked the question, “What should be the triage level of this patient according to the Emergency Severity Index (ESI)?” Patient information was presented in terms of age, gender, presenting complaint and duration, chronic disease status, and vital signs. The exact prompt template used was: “Patient: [age] year-old [gender], presenting with [complaint] for [duration]. Chronic diseases: [list/none]. Vital signs: Temperature [°C], Heart rate [bpm], Respiratory rate [/min], Blood pressure [mmHg], SpO2 [%]. What should be the triage level of this patient according to the Emergency Severity Index (ESI)?”

Nurse and physician assessments were conducted independently, and the ESI scores given by the nurses were kept confidential until the physician assessment was completed. Artificial intelligence assessments were conducted on the same day after the patient examination was completed.

### Statistical analysis

Data analysis was performed using SPSS 26.0. The research team presented descriptive statistics as numerical values which showed categorical data points as percentages and continuous data points as mean (SD) and median (IQR) values. Given the paired nature of the data, Cochran's *Q*-test was used for overall comparison of classification accuracy across evaluators, and McNemar's test with continuity correction was applied for pairwise comparisons. The researchers evaluated agreement between raters using Cohen's Kappa coefficient with 95% confidence intervals for interpretation at three levels of agreement: fair (0.21–0.40), moderate (0.41–0.60), and good (0.61–0.80). Weighted kappa was used for the ordinal ESI scale.

Ninety-five percent confidence intervals for all proportions were calculated using the Wilson score method. Mean absolute ESI error was calculated as the average absolute difference between evaluator and physician scores; severe mis-triage was defined as a difference of two or more ESI levels. The research used statistical methods to determine sensitivity and specificity values and PPV and NPV percentages with their 95% confidence intervals to identify patients who needed immediate medical care. The research used the Chi-square test with Bonferroni correction to analyze how different subgroups—including age groups, gender, and patients with chronic diseases—compared to each other. Statistical significance was set at *P* < 0.05.

### Ethical considerations

This study was approved by our hospital's Clinical Research Ethics Committee (Approval number: 4800, Date: 25/03/2025). Written informed consent was obtained from parents or legal guardians for all patients under 18 years of age. For adolescents aged 12–17 years, assent was also obtained in addition to parental consent, as required by the ethics committee. To protect patient privacy, personal data were anonymised and coded for analysis. Data security was ensured on encrypted computers and in databases accessible only to the study team. The study was conducted in accordance with the principles of the Declaration of Helsinki.

The commercial AI platforms were provided with all patient information after complete anonymization of all data before submission. The system received clinical vignettes which included age information and gender details and presenting complaints and vital signs and chronic disease status but it did not receive any personally identifiable information. Users needed to access the platforms by using web-based interfaces which required them to establish encrypted network connections with institutional computers. The data entered through web interfaces may serve model enhancement purposes as per OpenAI and xAI policies but the researchers determined that complete anonymization made re-identification risks extremely low. The research team received institutional approval to use the AI platform through their ethics committee approval process while following both the Turkish Personal Data Protection Law (KVKK) and GDPR regulations for anonymized data.

## Results

### Patient characteristics

A total of 1,505 paediatric emergency patients were included in the study. The mean age of the patients was 7.9 ± 5.1 years, with the youngest patient being a 1-month-old infant and the oldest patient being a 17-year-old and 11-month-old adolescent. In terms of gender distribution, there was a slight predominance of male patients; 52.1% of patients were male and 47.9% were female. The vast majority of admissions occurred on weekdays (82.7%), while weekend admissions accounted for 17.3% (Table 1).

**Table 1:** Demographic and clinical characteristics of the patients (*n* = 1,505).

Characteristics	*n* (%) or Mean ± SD (min–max)
Age (years)	7.9 ± 5.1 (0.1–17.9)
Sex	
– Female	721 (47.9)
– Male	784 (52.1)
Chronic disease status	
– None	1,339 (89.0)
– Present	166 (11.0)
• Asthma/Allergic asthma	31 (2.1)
• Epilepsy	17 (1.1)
• Allergic rhinitis	12 (0.8)
• Familial Mediterranean Fever (FMF)	11 (0.7)
• Hypothyroidism	7 (0.5)
• Type 1 Diabetes	7 (0.5)
• Attention Deficit Hyperactivity Disorder (ADHD)	8 (0.5)
• Other chronic diseases	73 (4.9)
Time of presentation	
– Weekdays	1,244 (82.7)
– Weekends	261 (17.3)
Presenting complaints*	
– Fever	431 (28.6)
– Cough	464 (30.8)
– Sore throat	390 (25.9)
– Vomiting/feeding disorder	191 (12.7)
– Musculoskeletal pain	68 (4.5)
– Dyspnea	65 (4.3)
– Diarrhea	63 (4.2)
– Headache	39 (2.6)
– Rash	37 (2.5)
– Ear pain	79 (5.2)
– Abdominal pain	49 (3.3)
– Eye redness	41 (2.7)
Vital signs	
– Temperature (°C)	36.8 ± 0.6
– Respiratory rate (/min)	24.5 ± 7.0
– SpO₂ (%)	99.1 ± 0.9
– Heart rate (/min)	102.4 ± 21.2
– Systolic BP (mmHg)	100.9 ± 11.0

Data are presented as *n* (%) or mean ± SD (min–max). SD, standard deviation; SpO₂, peripheral oxygen saturation; BP, blood pressure; FMF, familial Mediterranean fever; ADHD, attention deficit hyperactivity disorder. Multiple complaints may be present in a single patient.

*Multiple complaints may be present in a single patient.

Approximately one in ten patients had a chronic illness. The most common chronic diseases were asthma and allergic asthma, which were detected in 31 patients. These were followed by epilepsy (*n* = 17), allergic rhinitis (*n* = 12), familial Mediterranean fever (*n* = 11), type 1 diabetes (*n* = 7) and hypothyroidism (*n* = 7). Attention deficit hyperactivity disorder was seen in 8 patients, while the remaining 73 patients had other rare chronic diseases (Table 1).

Complaints presenting to the emergency department varied, and many patients had multiple complaints. The most common reasons for presentation were cough (30.8%), fever (28.6%) and sore throat (25.9%). Vomiting or feeding difficulties were seen in 12.7% of patients, muscle and joint pain in 4.5% of patients, and respiratory distress in 4.3% of patients. To a lesser extent, diarrhoea (4.2%), headache (2.6%), rash (2.5%), earache (*n* = 79; 5.2%), abdominal pain (*n* = 49; 3.3%) and eye redness (*n* = 41; 2.7%) were also recorded.

Vital signs were generally within normal limits; mean body temperature was 36.8°C, peripheral oxygen saturation was 99.1%, heart rate was 102.4 beats per minute, and systolic blood pressure was 100.9 mmHg (Table 1).

### Overall triage performance and agreement

Significant differences emerged between physicians, nurses, and artificial intelligence systems in triage assessments. Table 2 presents the overall triage performance and agreement analyses between different evaluators. Over-triage was defined as assignment of a lower ESI score than physician assessment, while under-triage was defined as assignment of a higher ESI score than physician assessment. Nurses correctly triaged 53.1% (95% CI: 50.6–55.6) of patients, while ChatGPT 4o achieved 76.1% (95% CI: 73.9–78.2) and Grok 3 achieved 47.0% (95% CI: 44.5–49.6) accuracy. The differences between evaluators were statistically significant (Cochran's Q = 275.68, *p* < 0.001), and all pairwise comparisons using McNemar's test were also significant (*p* < 0.001 for all pairs). When the agreement between evaluators was examined using Cohen's Kappa coefficient, ChatGPT 4o showed good agreement with physician evaluations [*κ* = 0.69 (95% CI: 0.66–0.72)], nurses showed moderate agreement [*κ* = 0.42 (95% CI: 0.39–0.45)], and Grok 3 showed fair agreement [*κ* = 0.31 (95% CI: 0.28–0.34)].

**Table 2:** Triage performance, agreement analyses, and critical patient identification metrics.

A. Overall performance and agreement							
Evaluator	Accuracy *n* (%, 95% CI)	Kappa (95% CI)	*p*	Critical Recognition *n*/*N* (%, 95% CI)	Over-triage *n* (%, 95% CI)	Under-triage *n* (%, 95% CI)	Severe Mis-triage^a^ *n* (%)
Nurse	799 (53.1, 50.6–55.6)	0.42 (0.39–0.45)	<0.001	48/129 (37.2, 29.4–45.8)	187 (12.4, 10.9–14.2)	519 (34.5, 32.1–37.0)	27 (1.8)
ChatGPT 4o	1,145 (76.1, 73.9–78.2)	0.69 (0.66–0.72)	<0.001	107/129 (82.9, 75.5–88.5)	137 (9.1, 7.7–10.7)	223 (14.8, 13.1–16.7)	11 (0.7)
Grok 3	708 (47.0, 44.5–49.6)	0.31 (0.28–0.34)	<0.001	126/129 (97.7, 93.4–99.2)	547 (36.3, 33.9–38.8)	250 (16.6, 14.8–18.6)	75 (5.0)

B. Detailed performance metrics for critical patient identification			
Metric	Nurse (95% CI)	ChatGPT 4o (95% CI)	Grok 3 (95% CI)
Sensitivity (%)	37.2 (29.4–45.8)	82.9 (75.5–88.5)	97.7 (93.4–99.2)
Specificity (%)	99.9 (99.6–100.0)	98.0 (97.2–98.6)	84.5 (82.5–86.3)
Positive predictive value (%)	98.0 (89.3–99.6)	79.9 (72.3–85.8)	37.2 (32.2–42.4)
Negative predictive value (%)	94.4 (93.1–95.5)	98.4 (97.6–98.9)	99.7 (99.2–99.9)
Mean absolute ESI error	0.49 ± 0.53	0.25 ± 0.45	0.58 ± 0.60

Percentages calculated from *n* = 1,505. CI, confidence interval; *N*, number of patients assessed as ESI-2 by physician (no ESI-1 cases observed). Cohen's Kappa interpretation: 0.21–0.40 = fair, 0.41–0.60 = moderate, 0.61–0.80 = good. Over-triage: assignment of a lower ESI score than physician assessment. Under-triage: assignment of a higher ESI score. Statistical comparisons: Cochran's *Q*-test for overall comparison (Q = 275.68, *p* < 0.001); McNemar's test for pairwise comparisons: Nurse vs. ChatGPT 4o (χ^2^ = 175.55, *p* < 0.001), Nurse vs. Grok 3 (χ^2^ = 10.34, *p* = 0.001), ChatGPT 4o vs. Grok 3 (χ^2^ = 222.86, *p* < 0.001).

^a^ Severe mis-triage: ESI difference ≥2 levels. Critical patient defined as ESI-2. 95% CIs calculated using Wilson score method.

### Critical patient identification

Critical patients were defined as ESI-2, as no ESI-1 cases were observed during the study period. There were also significant differences in the identification of these critically ill patients; while nurses correctly identified only 37.2% (95% CI: 29.4–45.8) of 129 patients classified as ESI-2 by physicians, ChatGPT 4o achieved 82.9% (95% CI: 75.5–88.5) and Grok 3 achieved 97.7% (95% CI: 93.4–99.2). The system needed users to decide between achieving high sensitivity levels and preserving high specificity levels based on its complete performance metrics which were essential for identifying critical patients. The test results from Grok 3 showed the highest sensitivity at 97.7% but it produced the lowest specificity at 84.5% (95% CI: 82.5–86.3) and positive predictive value at 37.2% (95% CI: 32.2–42.4). The nursing group achieved the highest specificity at 99.9% (95% CI: 99.6–100.0) and positive predictive value at 98.0% (95% CI: 89.3–99.6) yet their ability to detect cases was the lowest. ChatGPT 4o achieved a balanced performance with sensitivity of 82.9%, specificity of 98.0% (95% CI: 97.2–98.6), and positive predictive value of 79.9% (95% CI: 72.3–85.8) (Table 2B).

### Triage errors and Mis-triage patterns

When examining the magnitude of triage errors, the mean absolute ESI error was lowest for ChatGPT 4o (0.25 ± 0.45), followed by nurses (0.49 ± 0.53) and Grok 3 (0.58 ± 0.60). Severe mis-triage, defined as ESI difference of two or more levels, occurred in 27 patients (1.8%) for nurses, 11 patients (0.7%) for ChatGPT 4o, and 75 patients (5.0%) for Grok 3. Grok 3 led in terms of over-triage [36.3% (95% CI: 33.9–38.8)], while under-triage was most common among nurses [34.5% (95% CI: 32.1–37.0)] (Table 2A). The confusion matrices presented in Table 3 provide a detailed breakdown of classification patterns for each evaluator, demonstrating that Grok 3's high sensitivity came at the cost of substantial over-triage of ESI-3 patients into the ESI-2 category (172 cases).

**Table 3:** Confusion matrices for ESI classification by evaluator.

A. Nurse vs. Physician (Gold Standard)				
Nurse\Physician	ESI-2	ESI-3	ESI-4	ESI-5
ESI-2	48	1	0	0
ESI-3	62	160	15	3
ESI-4	19	285	298	276
ESI-5	0	5	40	293

B. ChatGPT 4o vs. Physician (Gold Standard)				
ChatGPT 4o\Physician	ESI-2	ESI-3	ESI-4	ESI-5
ESI-2	107	24	3	0
ESI-3	20	266	5	0
ESI-4	2	155	258	58
ESI-5	0	6	87	514

C. Grok 3 vs. Physician (Gold Standard)					
Grok 3\Physician	ESI-1	ESI-2	ESI-3	ESI-4	ESI-5
ESI-1	0	2	0	0	0
ESI-2	0	124	172	36	5
ESI-3	0	3	265	98	34
ESI-4	0	0	14	212	426
ESI-5	0	0	0	7	107

Rows represent evaluator assignments; columns represent physician (gold standard) assessments. Diagonal cells (shaded green) indicate correct classifications. No ESI-1 cases were identified by the physician during the study period. Total *n* = 1,505.

### ESI distribution and patient disposition

A strong correlation was found between ESI levels and patient disposition. The vast majority of patients in the study (95.7%) were discharged after outpatient treatment, while only 4.3% were admitted to hospital. Of these admissions, 46 were to wards and 18 to intensive care. Approximately half of the patients assessed as ESI-2 (46.5%) were admitted to hospital, while this rate dropped to 0.9% in the ESI-3 group. All patients with ESI-4 and ESI-5 levels were discharged. As noted above, there were no patients in the ESI-1 category during the study period (Table 4).

**Table 4:** Overall disposition and i'ts association with emergency severity Index (ESI) levels (*n* = 1,505).

Disposition	*n* (%)	Disposition by ESI level (Physician assessment)	Discharged *n* (%)	Admitted *n* (%)	Total
Overall					
– Discharged	1,441 (95.7)	ESI-1	–	–	0
– Admission (total)	64 (4.3)	ESI-2	69 (53.5)	60 (46.5)	129
• Ward admission	46 (3.1)	ESI-3	447 (99.1)	4 (0.9)	451
• ICU admission	18 (1.2)	ESI-4	353 (100.0)	0 (0.0)	353
		ESI-5	572 (100.0)	0 (0.0)	572

ESI, Emergency Severity Index; percentages in the “Overall” column are based on the entire cohort (*n* = 1,505), whereas percentages in the ESI section are calculated row-wise within each level; the association between ESI triage level and patient disposition was statistically significant (chi-square, *p* < 0.001).

**Figure 2:**
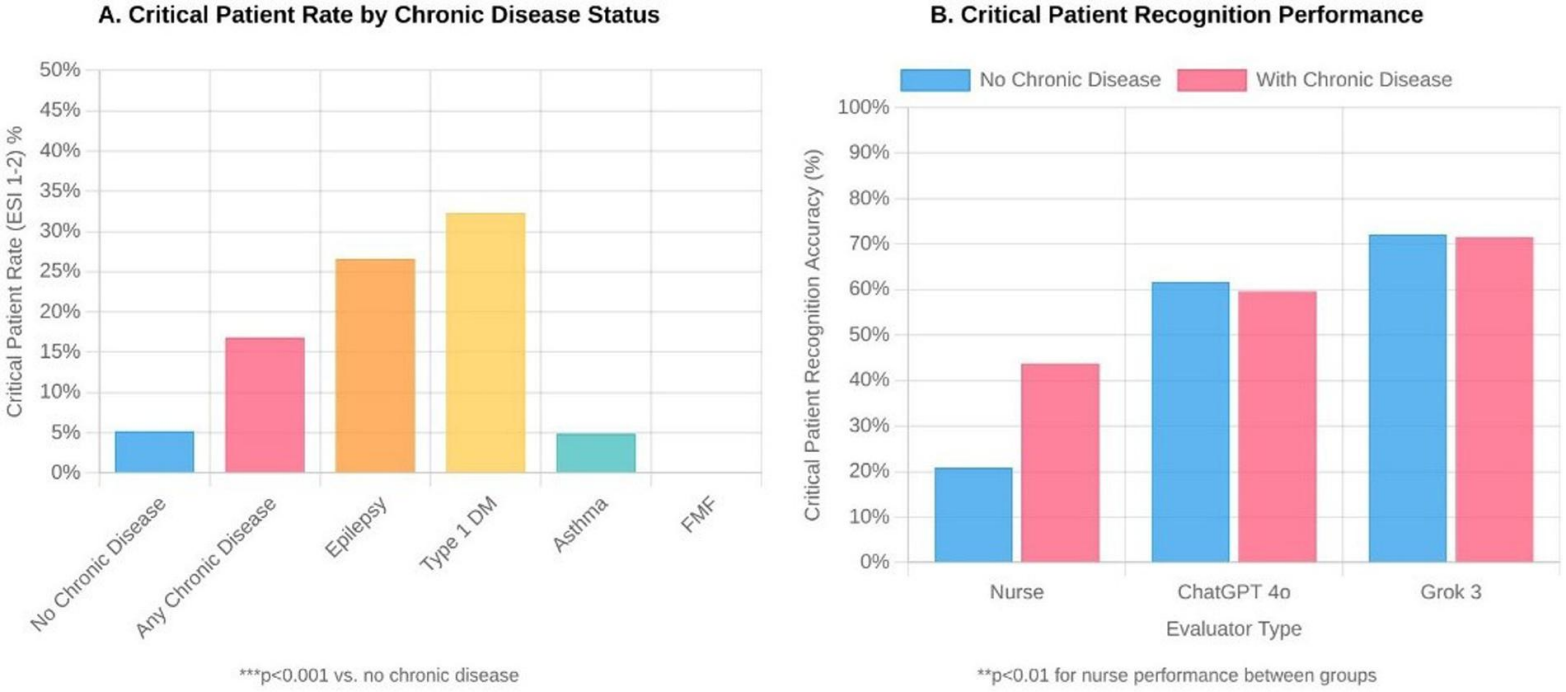
Distribution of emergency severity index (ESI) scores by evaluator type among 1,505 pediatric emergency department patients. **(A)** Critical patient rates (ESI 1-2) according to chronic disease status. **(B)** Critical patient recognition accuracy by evaluator type (nurse, ChatGPT 4o, and Grok 3), stratified by the presence or absence of chronic disease. ESI levels range from 1 (most urgent) to 5 (least urgent). Statistical significance (***p < 0.001) was determined using chi-square tests comparing differences between evaluators.

There were also significant differences between evaluators in terms of ESI distributions. According to physician assessments, 8.6% of patients were classified as ESI-2, 30.0% as ESI-3, 23.5% as ESI-4, and 38.0% as ESI-5. Nurses tended to assign more moderate ESI scores, while Grok 3 tended to place patients in more urgent categories. ChatGPT 4o's ESI distribution was closest to physician assessments (Figure 2).

### Performance by Age group and gender

When triage performance was examined by age group, the accuracy rate of nurses was lowest in the youngest age group (0–2 years), at 40.3% (95% CI: 34.0–46.9). ChatGPT 4o demonstrated consistent performance across all age groups, ranging from 72.6% (95% CI: 67.9–76.8) to 77.9% (95% CI: 73.4–81.8). Grok 3's performance remained below 50% across all age groups. No significant performance difference was found between evaluators in terms of gender (Table 5).

**Table 5:** Triage performance according to demographic and clinical subgroups (REVISED).

Subgroup	*n*	Nurse accuracy *n* (%, 95% CI)	ChatGPT 4o accuracy *n* (%, 95% CI)	Grok 3 accuracy *n* (%, 95% CI)	*p* *
Age groups					
0–2 years	221	89 (40.3, 34.0–46.9)	170 (76.9, 70.9–82.0)	106 (48.0, 41.5–54.5)	<0.001
2–5 years	390	220 (56.4, 51.5–61.2)	283 (72.6, 67.9–76.8)	191 (49.0, 44.0–53.9)	<0.001
6–11 years	519	293 (56.5, 52.2–60.7)	400 (77.1, 73.3–80.5)	245 (47.2, 42.9–51.5)	<0.001
12–17 years	375	197 (52.5, 47.5–57.5)	292 (77.9, 73.4–81.8)	166 (44.3, 39.3–49.3)	<0.001
Sex					
Female	721	386 (53.5, 49.9–57.1)	547 (75.9, 72.6–78.8)	341 (47.3, 43.7–50.9)	<0.001
Male	784	413 (52.7, 49.2–56.2)	598 (76.3, 73.2–79.1)	367 (46.8, 43.3–50.3)	<0.001
Chronic disease status					
Absent	1,339	706 (52.7, 50.0–55.4)	1,021 (76.3, 73.9–78.5)	622 (46.5, 43.8–49.1)	<0.001
Present	166	93 (56.0, 48.4–63.4)	124 (74.7, 67.6–80.7)	86 (51.8, 44.3–59.3)	<0.001

Accuracy percentages with 95% confidence intervals (Wilson score method) are based on comparison with physician assessment.

* Difference between evaluators was analyzed using Cochran's *Q*-test for multiple raters. McNemar's test was used for pairwise comparisons. All pairwise comparisons between evaluators were statistically significant (*p* < 0.001).

### Impact of chronic disease on triage performance

**Figure 3:**
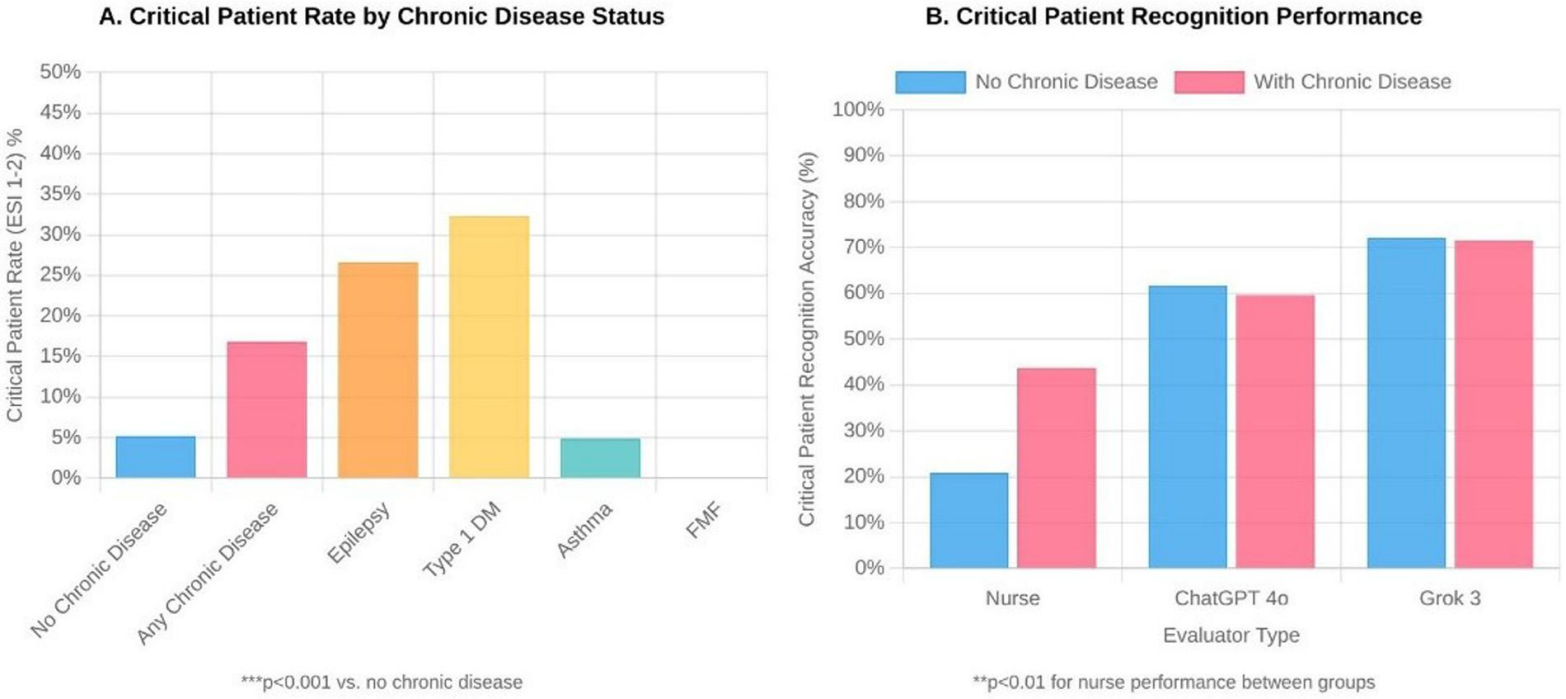
Impact of chronic disease on pediatric emergency triage (*n* = 1,505). Panel **A**: Critical patient rates (ESI 1–2) by chronic disease status. ***p < 0.001. Panel **B**: Critical patient recognition accuracy by evaluator type. **p < 0.01 for nurse performance between groups.

The effect of chronic disease on triage performance was striking. The proportion of critical (ESI-2) children with chronic disease was more than three times higher than that of children without chronic disease (22.3% vs. 6.9%, *p* < 0.001). Nurses were significantly more successful in identifying critically ill patients with chronic conditions [59.5% (95% CI: 43.5–73.7) vs. 28.3% (95% CI: 20.1–38.2), *p* < 0.01]. Critical patient rates were particularly high in patients with epilepsy and type 1 diabetes, at 35.3% and 42.9%, respectively (both *p* < 0.001 vs. no chronic disease). Both artificial intelligence systems maintained high critical patient recognition rates regardless of the presence of chronic disease (ChatGPT 4o: 83.7% vs. 81.1%; Grok 3: 97.8% vs. 97.3%) (Table 6, Figure 3).

**Table 6:** Effect of chronic disease on ESI distribution and critical patient recognition .

Chronic disease status	*n*	ESI 1–2 *n* (%)	ESI 3 *n* (%)	ESI 4–5 *n* (%)	Nurse *n*/*N* (%, 95% CI)	ChatGPT 4o *n*/*N* (%, 95% CI)	Grok 3 *n*/*N* (%, 95% CI)
Absent	1,339	92 (6.9)	387 (28.9)	860 (64.2)	26/92 (28.3, 20.1–38.2)	77/92 (83.7, 74.8–89.9)	90/92 (97.8, 92.4–99.4)
Present	166	37 (22.3)***	64 (38.6)	65 (39.2)	22/37 (59.5, 43.5–73.7)**	30/37 (81.1, 65.8–90.5)	36/37 (97.3, 86.2–99.5)
Common chronic diseases							
Asthma	31	2 (6.5)	12 (38.7)	17 (54.8)	2/2 (100.0, 34.2–100.0)	2/2 (100.0, 34.2–100.0)	2/2 (100.0, 34.2–100.0)
Epilepsy	17	6 (35.3)***	7 (41.2)	4 (23.5)	2/6 (33.3, 9.7–70.0)	5/6 (83.3, 43.6–97.0)	6/6 (100.0, 61.0–100.0)
Familial Mediterranean Fever (FMF)	11	0 (0.0)	5 (45.5)	6 (54.5)	–	–	–
Type 1 Diabetes Mellitus (DM)	7	3 (42.9)***	2 (28.6)	2 (28.6)	2/3 (66.7, 20.8–93.9)	2/3 (66.7, 20.8–93.9)	3/3 (100.0, 43.9–100.0)

DM, diabetes mellitus; FMF, familial mediterranean fever; *N*, number of patients assessed as ESI-2 by physician (no ESI-1 cases observed during the study period). ***p < 0.001, **p < 0.01 compared with patients without chronic disease (chi-square test for ESI distribution; Fisher's exact test for critical patient recognition due to small sample sizes). 95% confidence intervals calculated using Wilson score method. ESI, emergency severity index.

### Performance by presenting complaint

When performance was evaluated based on presenting complaints, ChatGPT 4o achieved a very high accuracy rate of 93.8% (95% CI: 85.2–97.6) in patients with respiratory distress. ChatGPT 4o also performed well for cough and sore throat complaints [76.3% (95% CI: 72.2–79.9) and 81.0% (95% CI: 76.8–84.6), respectively], while Grok 3 showed low accuracy rates for these complaints [35.6% (95% CI: 31.3–40.0) and 33.8% (95% CI: 29.3–38.7)]. Interestingly, Grok 3 performed better than the other evaluators for vomiting/feeding difficulties and diarrhoea complaints [80.6% (95% CI: 74.4–85.6) and 79.4% (95% CI: 67.8–87.5), respectively]. The performance of all evaluators decreased in patients with abnormal vital signs; nurses achieved an accuracy rate of 46.7% (95% CI: 37.4–56.2), ChatGPT 4o achieved 61.0% (95% CI: 51.4–69.7), and Grok 3 achieved 59.0% (95% CI: 49.5–68.0) (Table 7).

**Table 7:** Triage performance according to clinical characteristics .

Clinical feature	*n*	Nurse Accuracy *n* (%, 95% CI)	ChatGPT 4o Accuracy *n* (%, 95% CI)	Grok 3 Accuracy *n* (%, 95% CI)	*p* *
Presenting complaint					
Fever	431	229 (53.1, 48.4–57.8)	291 (67.5, 63.0–71.8)	252 (58.5, 53.8–63.0)	<0.001
Cough	464	224 (48.3, 43.8–52.8)	354 (76.3, 72.2–79.9)	165 (35.6, 31.3–40.0)	<0.001
Sore throat	390	222 (56.9, 52.0–61.7)	316 (81.0, 76.8–84.6)	132 (33.8, 29.3–38.7)	<0.001
Dyspnea	65	27 (41.5, 30.4–53.7)	61 (93.8, 85.2–97.6)	44 (67.7, 55.6–77.8)	<0.001
Vomiting/feeding disorder	191	63 (33.0, 26.7–39.9)	113 (59.2, 52.1–65.9)	154 (80.6, 74.4–85.6)	<0.001
Diarrhea	63	25 (39.7, 28.5–52.0)	35 (55.6, 43.3–67.2)	50 (79.4, 67.8–87.5)	<0.001
Vital signs					
Normal**	1,399	750 (53.6, 51.0–56.2)	1,080 (77.2, 74.9–79.3)	646 (46.2, 43.5–48.8)	<0.001
Abnormal***	105	49 (46.7, 37.4–56.2)	64 (61.0, 51.4–69.7)	62 (59.0, 49.5–68.0)	0.046

* Cochran's *Q*-test for multiple rater comparison. Multiple complaints may be present in a single patient.

** Normal: temperature <38°C and SpO₂ ≥ 95%.

*** Abnormal: temperature ≥38°C or SpO₂ < 95%. 95% confidence intervals calculated using Wilson score method.

### Confusion matrix analysis

The confusion matrices (Table 3) show the overall classification patterns which each evaluator used during their assessment process. The nursing staff made ESI-3 to ESI-4 under-triaging as their primary mistake which occurred 285 times. The staff showed a pattern of assigning lower acuity scores to patients. ChatGPT 4o showed the most balanced classification results because its prediction mistakes occurred at similar rates throughout the test; ESI-3 patients received the most incorrect assignments when they were placed in the ESI-4 category (155 cases). Grok 3 demonstrated a specific pattern which involved the consistent upgrading of patients from ESI-3 to ESI-2 and from ESI-5 to ESI-4. Notably, Grok 3 was the only evaluator to assign ESI-1 scores (2 cases), both of which were for patients classified as ESI-2 by physicians. The pattern of over-triage explains why Grok 3 shows 97.7% sensitivity in detecting critical patients yet its positive predictive value remains at 37.2% because 213 out of 337 patients (63.2%) who received critical flags turned out to be non-critical according to physician evaluation.

## Discussion

We evaluated how well modern artificial intelligence systems perform in pediatric emergency department triage evaluations relative to experienced nurses and specialist physicians. The artificial intelligence system achieved correct decisions in 75% of cases when nurses reached only 50% accuracy. The artificial intelligence systems demonstrated exceptional ability to detect patients who needed immediate critical care. The artificial intelligence system proved better than nurses at detecting critical cases because it correctly identified almost all such cases but nurses only found one-third of them. The artificial intelligence system demonstrated a different performance pattern when assessing children with ongoing medical conditions because nurses achieved double the accuracy rate with this specific patient group. The analysis revealed distinct age-related performance patterns because artificial intelligence maintained steady results across all age groups yet nurses struggled most with infant cases.

The use of physicians as the gold standard for triage assessment needs further explanation. The research team conducted physician ESI assessments right after nurse triage was initiated, within 15–30 min after patients arrived at the facility. The physicians who made ESI assignments did not have access to laboratory results and imaging studies or treatment responses, which reduced the chance of verification and incorporation bias that would occur when *post hoc* clinical data affect the reference standard. The routine triage work of nurses contrasts with physician assessments because doctors bring full clinical expertise to their evaluations while working under minimal time constraints and workflow limitations. The final clinical decision which determines patient care routes and actual treatment paths makes physicians the best reference standard for research purposes. The approach follows established triage validation research which uses physician consensus to evaluate accuracy.

Our results indicated ChatGPT 4o achieved better results than nurses since it reached 76.1% (95% CI: 73.9–78.2) accuracy while nurses only achieved 53.1% (95% CI: 50.6–55.6). The difference between evaluators was statistically significant using Cochran's *Q*-test (Q = 275.68, *p* < 0.001), and pairwise McNemar's tests confirmed significant differences between all evaluator pairs (*p* < 0.001). This finding is consistent with the work of Colakca and colleagues in a real emergency department setting; they also reported ChatGPT-4 achieving a 76.6% accuracy rate (8). Similarly, ChatGPT 4o's good agreement with physician evaluations [*κ* = 0.69 (95% CI: 0.66–0.72)] exactly matches the kappa value found by Lu et al. in GPT-4-based systems (9). Our study results showed moderate agreement between nurses [*κ* = 0.42 (95% CI: 0.39–0.45)] which matches findings in existing research. A study included in Kaboudi et al.'s meta-analysis showed human and ChatGPT-3.5 assessment agreement at a similar moderate level (*κ* = 0.320) (10). Furthermore, the same meta-analysis showed that ChatGPT-3.5 had an accuracy rate of 63% in triage (10). Lu and colleagues also reported that ChatGPT-4 yielded consistent results with physicians in ESI distribution, supporting our finding that ChatGPT 4o showed an ESI distribution close to physician assessments (9).

The superior performance of ChatGPT 4o compared to Grok 3 (76.1% vs. 47.0% accuracy) likely stems from several factors. The medical literature and clinical scenarios training of ChatGPT-4 received special optimization from OpenAI for healthcare use. The training data of ChatGPT contains a wider range of medical case studies and triage scenarios. The multi-layered attention mechanisms of ChatGPT might handle the intricate clinical variable relationships that appear in triage decisions more effectively. The system performance results are consistent with what researchers have found when they compared different AI system designs for medical work. The research by Lansiaux and his team evaluated three AI systems that applied Natural Language Processing (NLP), Large Language Models (LLMs), and Joint Embedding Predictive Architecture (JEPA) to measure their ability to predict triage outcomes. The research showed that LLM-based architecture produced the highest results for triage prediction because it reached an F1-score of 0.900 and an AUC-ROC of 0.879, which surpassed both NLP (F1: 0.618, AUC: 0.642) and traditional nurse triage (F1: 0.303, AUC: 0.776) in clinical decision-making tasks (11). The training data of Grok 3 likely emphasized sensitivity over specificity because its over-triage rate reached 36.3% (95% CI: 33.9–38.8) which indicates different risk tolerance in its training objectives. The training process with Reinforcement Learning from Human Feedback (RLHF) likely created this pattern because developers of the model during its development phase focused on safety-oriented feedback that prioritized sensitivity over specificity through “safety rails” design principles in AI development. The system demonstrates how Grok 3 tended to assign patients to higher acuity groups because it learned to avoid missing severe cases by making additional false-positive patient identifications. The results from the confusion matrix analysis confirm this finding because Grok 3 moved 172 ESI-3 patients to ESI-2 status and 426 ESI-5 patients to ESI-4 status, which indicates that the system performed over-triage in a consistent manner instead of making arbitrary mistakes.

We demonstrated that artificial intelligence systems outperform human nurses in detecting critical patient conditions because Grok 3 reached 97.7% sensitivity (95% CI: 93.4–99.2) and ChatGPT 4o reached 82.9% sensitivity (95% CI: 75.5–88.5) while nurses correctly identified only 37.2% (95% CI: 29.4–45.8) of cases. The evaluation of these two tests needs careful analysis because it involves a comprehensive diagnostic performance assessment. The high sensitivity of Grok 3 resulted in low specificity, which reached 84.5% (95% CI: 82.5–86.3) and a positive predictive value, which was 37.2% (95% CI: 32.2–42.4). The system incorrectly identified 63% of patients who received critical flags as being actually non-critical. The nursing team reached the highest specificity rate of 99.9% (95% CI: 99.6–100.0) and a positive predictive value of 98.0% (95% CI: 89.3–99.6), which demonstrated their ability to correctly identify critical patients in nearly all situations. ChatGPT 4o demonstrated the best performance balance through its high sensitivity of 82.9% and its high specificity of 98.0% and positive predictive value of 79.9%, which indicates it achieves the best combination of detection accuracy and precision. The mean absolute ESI error results demonstrate that ChatGPT 4o outperforms the other two systems because it achieved a value of 0.25 ± 0.45 compared to 0.49 ± 0.53 for nurses and 0.58 ± 0.60 for Grok 3. The lowest rate of severe mis-triage occurred at 0.7% for ChatGPT 4o when patients received differences of more than two ESI levels compared to nurses at 1.8% and Grok 3 at 5.0%. Research indicates that ML/AI systems working with ESI protocols decrease medical errors and enhance emergency patient detection accuracy because AI systems demonstrate 0.9% mistriage rates whereas traditional methods reach 1.2% (4). The results match earlier research which demonstrates nurses struggle to detect patients who need urgent medical care. The study by Huabbangyang and colleagues showed nurses reached a 69.1% total accuracy in ESI-based triage but their ability to detect critical patients at ESI levels 1–2 remained poor (12, 13). The PAT (Paediatric Assessment Triangle) tool enabled nurses to identify critical patients with a high success rate of 93.24% according to Ma and colleagues (14). This discrepancy highlights the importance of the assessment tool used and training. Grok 3's high over-triage rate of 36.3% indicates that the system is overly cautious. Viana and colleagues also reported an over-triage rate of around 12%–14% in traditional triage rules, which is similar to our nursing group's over-triage rate of 12.4% (95% CI: 10.9–14.2) (15). The high under-triage rate of 34.5% (95% CI: 32.1–37.0) observed among nurses indicates a serious safety issue. Hinson and colleagues reported an undertriage rate of 18.4% in ESI 4–5 patients, while Huabbangyang and colleagues reported an overall undertriage rate of 4.9% (12, 13). Our finding is well above these values and may reflect the unique challenges of the paediatric population. The hospitalisation of 46.5% of ESI 1–2 (specifically ESI-2, as no ESI-1 cases were observed) patients confirms that this group is truly critical and is consistent with the literature.

We confirmed that children with chronic illnesses had a critical patient rate of 22.3% which exceeded the 6.9% rate found in healthy children (*p* < 0.001) according to existing medical literature. The research by Al Zamel and colleagues showed that children with chronic illnesses received elevated triage priority levels and needed more critical care services (16). The results showed nurses achieved better results in detecting critical illness among children with chronic diseases [59.5% (95% CI: 43.5–73.7) vs. 28.3% (95% CI: 20.1–38.2) in children without chronic diseases, *p* < 0.01] yet ChatGPT 4o demonstrated comparable accuracy [81.1% (95% CI: 65.8–90.5) vs. 83.7% (95% CI: 74.8–89.9)] in this specific patient group. Grok 3 maintained near-perfect sensitivity regardless of chronic disease status (97.3% vs. 97.8%). The results indicate that nurses' clinical expertise helps them better evaluate complex chronic cases yet AI systems perform with more reliability and consistency in general. The nurses' assessment process becomes more thorough when they detect chronic diseases in patients. The results of training programs show that nurses who receive specialized triage education achieve a 94% knowledge level in complex case assessment and an 88% level in clinical decision-making (17). The high critical patient rates among patients with epilepsy (35.3%) and type 1 diabetes (42.9%) demonstrate the potential for acute decompensation in these medical conditions (both *p* < 0.001 vs. no chronic disease). The research by Sax and colleagues showed that patients with multiple health conditions face higher risks of receiving inadequate triage which results in delayed essential medical care (18). The artificial intelligence systems maintain their performance levels without any influence from chronic disease presence because they process standardized clinical parameters consistently. The research by Almulihi and colleagues demonstrated that AI-based triage systems achieved high precision in detecting critical patients regardless of their chronic disease status and machine learning algorithms successfully forecast hospitalization requirements with high precision for asthma patients and other chronic diseases (19). The nurses' 100% accuracy rate in critical patient recognition among asthmatic patients likely results from their deep understanding of this particular patient group and the small sample size (*n* = 2 critical patients with asthma).

The research confirms ESI should stay as the core triage framework because AI systems would function as additional tools to improve current protocols. The emergency department has proven the effectiveness of ESI as a validated system which remains reliable for its purpose (3). The combination of AI systems with ESI protocols through clinical decision support tools leads to better triage accuracy and lower mistriage rates according to studies which show KATE™ achieves 75.7% accuracy while nurses reach 59.8% accuracy (4). The research indicates that pediatric emergency triage benefits most from using AI to enhance the existing ESI system instead of creating separate AI-based triage systems.

The deployment of LLMs for clinical triage operations requires evaluation of multiple obstacles which extend beyond their ability to make accurate diagnoses. The research by Lansiaux and his team evaluated large language models for emergency department triage, which exposed multiple critical barriers that block their implementation in clinical practice. The AI systems used in emergency medicine process various data sources to create predictive results, but their accuracy remains unstable because they operate slowly, produce irregular output formats, do not integrate well with electronic health records, and require backup systems to handle ambiguous medical situations (20). The web-based access to AI systems in our research study produced delays, which make them inappropriate for emergency departments that need to process many cases. The black box operation of LLM systems prevents medical personnel from executing their professional duties because they cannot view the system's decision-making mechanisms. The system produces triage suggestions that clinicians cannot understand because they cannot view the underlying decision-making system that created these recommendations. The future development of this system should implement API-based integration, which will produce explicit reasoning outputs and provide standardized confidence metrics and defined procedures for handling cases with unclear results. The workflow integration must also handle medicolegal matters related to responsibility when AI-based triage systems produce adverse results from their decisions.

The 0–2 age group proved to be the most difficult for nurses to triage correctly since they reached only 40.3% (95% CI: 34.0–46.9) accuracy in this age range. The current triage systems according to Hopman and colleagues assign low priority to children between 0 and 2 years old and their sensitivity reaches only 50% when using MTS especially for infants under three months (21). The performance of ChatGPT 4o remained steady at 72.6% (95% CI: 67.9–76.8) to 77.9% (95% CI: 73.4–81.8) across every age group which shows artificial intelligence functions without age-related dependencies. The research by Abdul-Hafez and colleagues shows that artificial intelligence models excel at minimizing performance differences that stem from age-related factors (22). The artificial intelligence system ChatGPT 4o achieved a remarkable 93.8% (95% CI: 85.2–97.6) success rate when diagnosing respiratory distress cases. AI systems show better performance in particular clinical situations such as sepsis detection and bacteremia risk evaluation and critical care requirement forecasting through deep learning models which outperform emergency physicians with 0.95 sensitivity and 0.90 accuracy in critical outcome prediction (4). The artificial intelligence model according to Ellertsson and colleagues successfully detected patients at high risk who presented with acute respiratory symptoms (23). The artificial intelligence model Grok 3 achieved good results in vomiting/feeding disorder and diarrhoea complaints [80.6% (95% CI: 74.4–85.6) and 79.4% (95% CI: 67.8–87.5), respectively] but showed poor accuracy when assessing cough and sore throat symptoms [35.6% (95% CI: 31.3–40.0) and 33.8% (95% CI: 29.3–38.7), respectively]. Different artificial intelligence models demonstrate varying levels of success when evaluating different types of complaints. All evaluators demonstrated decreased performance when assessing abnormal vital signs with accuracy rates of 46.7% (95% CI: 37.4–56.2) for nurses, 61.0% (95% CI: 51.4–69.7) for ChatGPT 4o, and 59.0% (95% CI: 49.5–68.0) for Grok 3. The research by Hwang and colleagues showed that AI-CAD failed to outperform traditional methods when detecting abnormal findings in acute respiratory symptoms (24). The results show that all evaluators struggle with abnormal vital parameters which requires additional development in this area.

### Limitations

Our study has several limitations. The research results from our single-center study during a one-month period might not apply to other medical facilities, patient groups, or different times of year. The AI systems evaluated text-based clinical information, but they did not receive visual data, which nurses use to observe patients and interact with their families. The reference standard lacks formal inter-rater reliability assessment between the four physicians, which could have resulted in inconsistent measurement results. Future research should employ ordinal mixed-effects models for a more comprehensive analysis in addition to our current use of McNemar's and Cochran's *Q*-tests for paired comparisons. The AI evaluation process ran only once for each case because LLM output randomness may produce different results when performing multiple assessments. The system processed all transmitted data through full anonymization to meet all requirements of local privacy regulations. Future commercial AI platforms need to create solutions that meet data governance standards to operate their business operations. The study lacks ESI-1 cases because we excluded ambulance patients from the analysis, which restricts the findings with respect to the most severe emergency situations.

### Strengths

Despite these limitations, our study has notable strengths including prospectively collected real-world emergency department data, blinded and independent assessments between nurses and physicians, and the simultaneous evaluation of two distinct AI models. Future studies should conduct their investigations at different locations, and researchers must track participants throughout longer periods to fully assess AI-based triage system performance, including their error rates.

### Conclusion

The research shows that artificial intelligence systems work well as supportive tools to assist pediatric emergency triage operations. The AI system ChatGPT 4o achieved better results than nurses in detecting severe medical cases and maintained reliable performance across all patient age ranges. The AI systems showed varying levels of case detection ability, which affected their accuracy in identifying truly critical patients. The systems achieved higher detection rates, but this came at the cost of increased patient over-triage. The nursing assessment of children with chronic conditions produced better results because nurses utilize their clinical expertise, which AI systems currently lack. ChatGPT 4o demonstrated the most balanced system performance among all evaluated systems, which makes it an appropriate choice for clinical decision support applications. The research results confirm that AI should function as an additional tool that nurses can use but should not replace their professional nursing abilities. The optimal approach to paediatric emergency triage should integrate AI-based objective assessment with the clinical expertise and observational skills of trained nurses.

## Data Availability

The raw data supporting the conclusions of this article will be made available by the authors, without undue reservation.
